# Oxidative dissolution of Cr-doped UO_2_ nuclear fuel

**DOI:** 10.1038/s41529-023-00347-4

**Published:** 2023-04-07

**Authors:** Hannah Smith, Théo Cordara, Clémence Gausse, Sarah E. Pepper, Claire L. Corkhill

**Affiliations:** NucleUS Immobilisation Science Laboratory, Department of Materials Science and Engineering, The University of Sheffield, Sheffield, UK

**Keywords:** Corrosion, Nuclear chemistry

## Abstract

Alternative UO_2_ nuclear fuels, incorporating Cr as a dopant, are currently in use in light–water reactors. Dissolution experiments using Cr-doped UO_2_, performed as a function of Cr content in a simplified groundwater solution and under oxic conditions, established that the addition of Cr to the UO_2_ matrix systematically reduced the normalised dissolution rate of U at 25 and 40 °C. This effect was most notable under dilute solution conditions, and is the result of galvanic coupling between Cr and U, resulting from the presence of Cr^2+^ in the UO_2_ matrix, as corroborated by activation energy determination. Under conditions of solution saturation, where schoepite ((UO_2_)_8_O_2_(OH)_12_·(H_2_O)_12_) and Na_2_U_2_O_7_·6H_2_O were identified as secondary phases, the rate of U dissolution was invariant with Cr content. Moreover, at 60 °C, the trend was reversed and the rate of U dissolution increased with increasing Cr content. Under these conditions, other factors, including U solubility or bicarbonate-surface interactions, exert a stronger influence on the U dissolution kinetics than Cr. Increased grain size, a feature of Cr-doped UO_2_ fuel, was also found to reduce the normalised dissolution rate of U. In establishing the mechanisms by which Cr dopants influence UO_2_ fuel dissolution, it can be concluded that, overall, Cr-doped UO_2_ nuclear fuel possesses similar dissolution kinetics to undoped UO_2_ fuel, giving confidence for its eventual disposal in a geological facility.

## Introduction

The current option for the final disposal of spent UO_2_ nuclear fuel is a burial in an engineered geological disposal facility. This will consist of a multi-barrier containment system, designed for the retention of radionuclides and mitigation against radioactive elements reaching the biosphere. While engineered to promote minimal degradation, there is expected to be a point in time at which groundwater will reach spent fuel and facilitate dissolution processes.

The dissolution of spent UO_2_ fuel has been well established through many years of investigation of dissolution under anoxic, reducing and oxic conditions, e.g. refs. ^1–3^. For the latter, dissolution rates are quoted to range from between 1 and 7 mg m^−2^ d^−1^, with the highest rates obtained in the presence of carbonate ions due to the propensity of U^6+^ to complex with CO_3_^2−^ to form soluble species^3,4^. However, the development and use of alternative UO_2_ fuels, adapted by doping with elements such as Cr_2_O_3_ to enhance in-reactor performance^5–7^, necessitates further investigation to understand whether dissolution rates are influenced by doping.

Although complex in final composition and microstructure post-fission^4^, dissolution of the UO_2_ matrix is understood to be the rate-determining step of spent fuel degradation, which proceeds *via* the oxidation of U^4+^O_2_ to soluble U^6+^O_2_^2+^ species. This mechanism is governed by the availability of oxygen to diffuse through the lattice, enhanced by the presence of oxygen vacancies (O_v_)^8^. In the new alernative nuclear fuel, Cr-UO_2_, it has been shown that Cr doping of UO_2_ results in the substitution of Cr^2+^ onto U^4+^ sites in the lattice, with the concurrent formation of O_v_ and U^5+^ to form a compound with a stoichiometry of $\left( {\left( {U_{1 - x}^{4 + }U_x^{5 + }} \right)_{1 - y}Cr_y^{2 + }} \right)O_{2 - \frac{y}{2}}$ ^9^. As such, the presence of O_v_ and U^5+^ defect species should tend to increase the oxidative dissolution of doped UO_2_, which may reduce durability in a geological disposal facility when compared with conventional, undoped, UO_2_ fuel types.

However, a recent study focused on Cr-doped UO_2_ dissolution behaviour indicated the opposite behaviour, that the presence of Cr *decreased* the dissolution rate of U relative to pure UO_2_^10^. The authors postulated that the high pH conditions of their study (cementitious water, pH 13.5 and bicarbonate water, pH 9) caused the surface of Cr-UO_2_ to be more resistant to oxidation than UO_2_, or it otherwise prevented the release of U^6+^ species^10^. A slower rate of U dissolution compared with undoped UO_2_ was also reported for the dissolution of commercially fabricated Cr/Al-doped UO_2_ under oxidative conditions (H_2_O_2_), although it was stated that the behaviour may be due to differences in the surface area between samples, rather than the presence of Cr^11^. These authors were unable to establish a mechanism to satisfactorily explain why the addition of Cr influenced the UO_2_ behaviour.

It may be possible to partly explain these observations by reference to other doped systems, for example, a reduction in U dissolution rate compared to pure UO_2_ was also observed for trivalent Gd^3+^-doped UO_2_^12–15^. At room temperature and in bicarbonate solution (pH 7– 8.5), the dissolution rate of U was an order of magnitude lower for Gd-doped UO_2_, while a drop of almost two orders of magnitude was observed at 50 and 75 °C, thought to confirm a 'matrix stabilisation effect' of Gd in UO_2_^15^. It was postulated that stabilisation was conferred by a decrease in the degree of UO_2_ oxidation (U^4+^O_2_ to U^4+/5+^_4_O_9_) due to the substitution of U^4+^ cations for Gd^3+^ and potential formation of MO_8_-type defect clusters^11,14^. The same behaviour was observed for Dy^3+^-doped UO_2_^15,16^. MO_8_-type defect clusters have not been reported for Cr-doped UO_2_, therefore, another mechanism must exist.

The addition of Cr to UO_2_ fuel is known to result in an enlarged grain size when compared to standard UO_2_^5–7^. Grain boundaries have been shown to influence the dissolution behaviour of UO_2_ and spent fuel analogue materials including CeO_2_ and ThO_2_ and Ln-doped CeO_2_^17–24^. Assessment of the transformation of grain boundaries during dissolution found that these features contribute significantly to the release of U (or U analogue element). It was hypothesised that the concentration of O_v_ defects at grain boundaries creates high-energy reactive surface sites for dissolution to initiate^18^. This suggests that a decrease in the quantity of grain boundaries, which is associated with a larger grain size, as observed for Cr-doped UO_2_, could reduce the number of energetically reactive sites and, thus, reduce dissolution rates.

To establish the influence of Cr as a dopant on the dissolution kinetics of UO_2_, and to underpin the mechanisms that govern U release from Cr-doped UO_2_, semi-static oxic dissolution experiments in a simplified bicarbonate groundwater solution were performed. Two possible mechanisms were evaluated: (1) Cr-induced changes to the crystal chemistry and (2) microstructure. For (1), materials were prepared using a range of Cr-dopant concentrations, above and below the solubility limit of Cr in UO_2_, and the dissolution kinetics of U were determined as a function of Cr content and dissolution temperature. These materials were prepared such that there was a fixed grain size, to enable isolation of the chemical effects of Cr on the dissolution rate from those of the microstructure. For (2), materials were prepared at different sintering temperatures, producing pellets with varying grain size, to quantify the extent to which microstructure influences the dissolution of Cr-doped UO_2_.

## Results and discussion

### Effect of Cr concentration on the dissolution of UO_2_

Pellets sintered at 1700 °C were used to study the effect of Cr content and dissolution temperature on the dissolution rate of U, independently from microstructure effects.

**Fig. 1:**
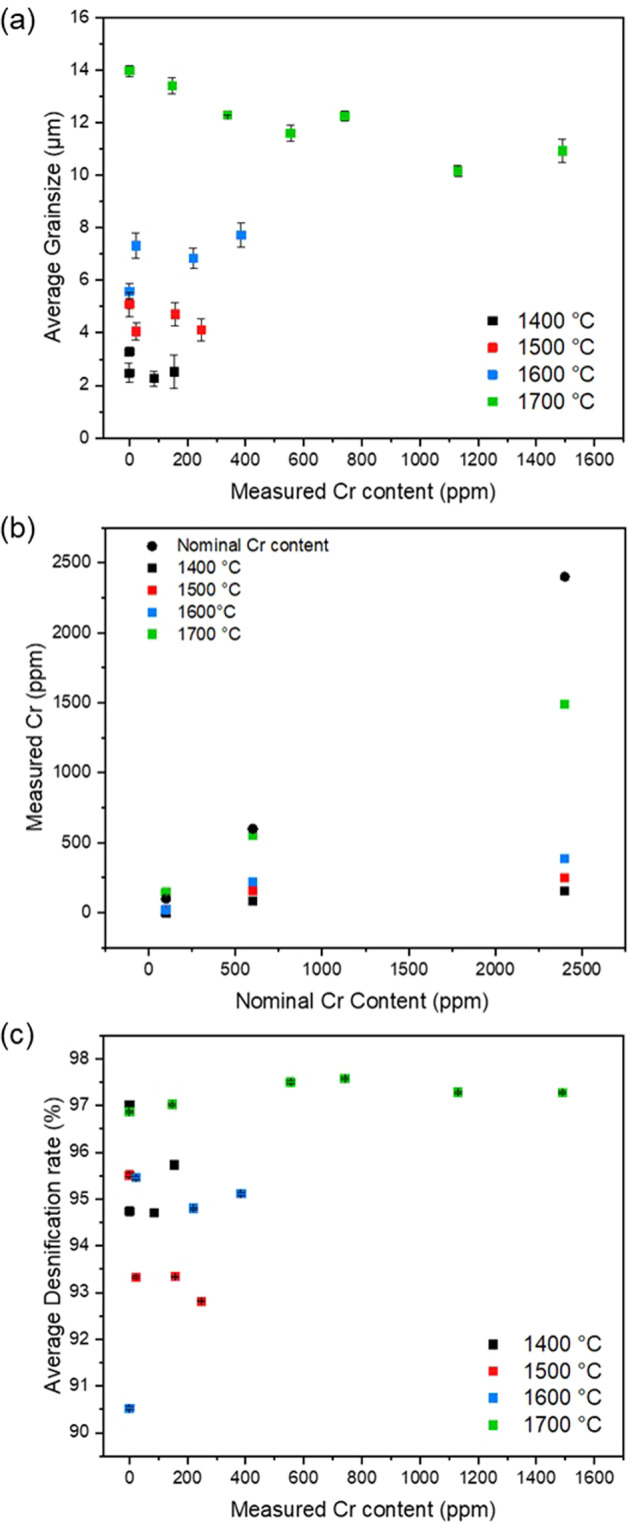
Microstructural changes and measured Cr content in Cr-doped UO_2._ **a** Grain size as a function of Cr content; **b** the measured Cr content of Cr-doped UO_2_ sintered at different temperatures (1400–1700 °C); and **c** Archimedes density. Error bars represent one standard deviation of triplicate measurements.

The microstructure and defect chemistry of the Cr-doped UO_2_ materials utilised in the dissolution experiments was detailed fully by Smith et al. ^9^. Briefly, for all concentrations of Cr-doped UO_2,_ the sintered pellets presented a homogenous distribution of Cr^2+^ within the UO_2_ matrix, charge compensated by O_v_ defects. Above the solubility limit of Cr within UO_2_ (700–1200 ppm)^25–28^, precipitates of Cr were observed to reside within grain boundaries, hypothesised to be Cr^3+^_2_O_3_^9,29^. When sintered at 1700 °C, the presence of these precipitates in Cr-doped UO_2_ resulted in a reduction in grain size when compared to undoped UO_2_ (Fig. 1a), due to the grain boundary pinning effect of precipitates, which inhibit diffusion and grain growth^30^.

The intentional absence of large grains (>30 μm), as observed in industrially synthesised Cr-doped UO_2_^6^, is a direct result of sintering in a fully reducing atmosphere; it is well understood that a controlled oxygen potential of the sintering atmosphere can promote grain growth in Cr-doped UO_2_ materials^26^. Indeed, the grain size did not vary significantly with Cr content when sintered at 1700 °C (Fig. 1a). Complete digest of the pellets sintered at 1700 °C showed that the Cr content was below the expected, nominal, Cr concentration (Fig. 1b), which is attributed to volatilisation at the high sintering temperature^9,31,32^.

**Fig. 2:**
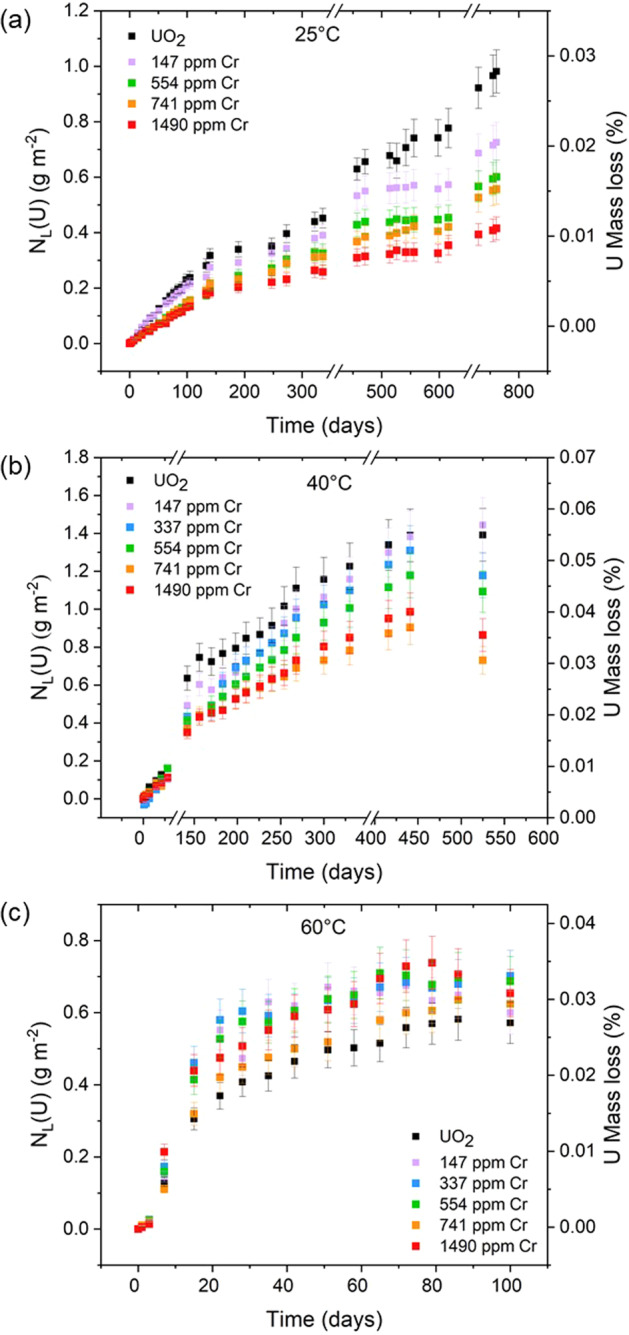
Normalised mass loss of U (N_L_(U)) from Cr-doped UO_2_ dissolved in bicarbonate solution. Data derived from dissolution experiments performed at **a** 25 °C, **b** 40 °C and **c** 60 °C, using materials sintered at 1700 °C. Error bars represent one standard deviation of triplicate measurements.

**Fig. 3:**
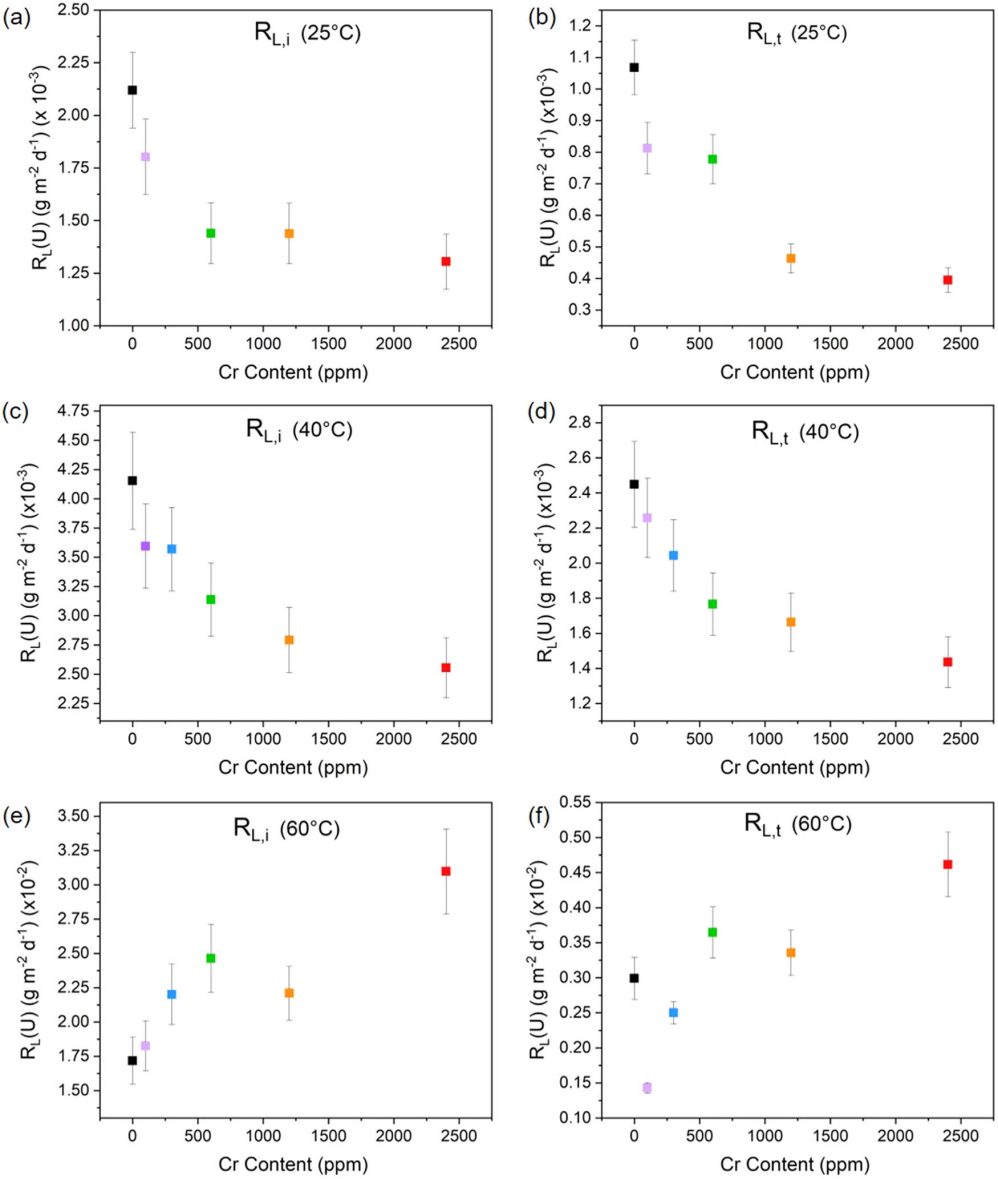
Normalised dissolution rates of U of Cr-doped UO_2_ dissolved in bicarbonate solution in the initial (R_L,i_) and transitional (R_L,t_) regimes. Data derived from dissolution experiments performed at **a**, **b** 25 °C, **c**, **d** 40 °C and **e**, **f** 60 °C, using materials sintered at 1700 °C. Error bars represent one standard deviation of triplicate measurements.

**Fig. 4:**
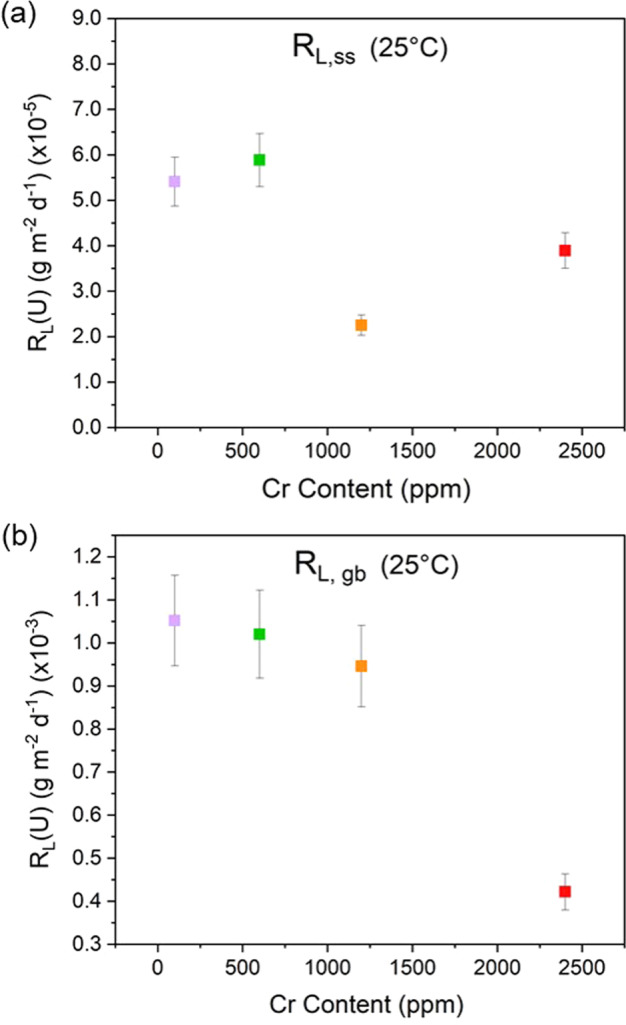
Normalised dissolution rates of U of Cr-doped UO_2_ dissolved in bicarbonate solution at 25 °C. Showing **a** the steady state (R_L,ss_) and **b** the grain boundary-influenced (R_L,gb_) regimes. Data were acquired using materials sintered at 1700 °C. Error bars represent one standard deviation of triplicate measurements.

The normalised mass loss of U, N_L_(U) (g m^−2^), at 25, 40 and 60 °C as a function of time (Fig. 2a–c) exhibited several distinct regimes of normalised dissolution rate, R_L_(U) (g m^−2^ d^−1^), as shown in Figs. 3, 4. The interpretation of these regimes was guided predominantly by the concentration, in mol L^−1^, of U leached from undoped UO_2_, which is reported to reach a solubility limit with respect to schoepite, of around 10^−5^ mol L^−1^, in saline, carbonate-containing solutions under oxic conditions^33^. The presence (or absence) of secondary phases was also used to support the identification of the regimes, as discussed later. The common regimes for all temperatures were interpreted as being: (1) R_L,i_, reflecting an initial dissolution rate where solution conditions were dilute and saturation effects were not observed, i.e. where U concentrations were ~10^−7^ mol L^−1^ (Fig. 3a, b); (2) R_L,t_, describing a transitional stage of dissolution where the effects of solution saturation commenced and the dissolution rate of U began to decrease, i.e. where U concentrations were ~10^−6^ mol L^−1^ (Fig. 3c, d); and (3) R_L,ss_ where the thermodynamic, solubility-related effects of elements in solution were observed (steady state), i.e. where U concentrations were ~10^−5^ mol L^−1^ and a plateau, or drop, in NL_(U)_ was observed (Fig. 4a).

For Cr-doped materials dissolved at 25 °C only, an additional regime, R_L,gb_, where the normalised dissolution rate of U increased at later time points (Figs. 2a, 4b) was observed. Dissolution of UO_2_ in acidic media has attributed this apparent increase in dissolution to an increase in the surface area associated with the dissolution of grain boundaries^23,34–36^. Table 1 details the time period (days) of each regime for materials dissolved at each temperature, Table 2 gives the absolute dissolution rate values in g m^−2^ d^−1^ and Supplementary Table 1 in mol L^−1^ d^−1^.

**Table 1:** Time periods selected for the determination of the normalised dissolution rate regimes.

		Time period (days)					
Temperature (°C)	Rate regime	UO_2_	147 ppm Cr	337 ppm Cr	554 ppm Cr	741 ppm Cr	1490 ppm Cr
25	R_L,i_	0–133	0–133	-	0–105	0–133	0–140
	R_L,t_	140–761	140–457	-	133–457	140–514	189–471
	R_L,ss_	-	471–598	-	471–598	471–598	514–598
	R_L,gb_	-	616–761	-	616–761	616–761	616–761
40	R_L,i_	0–183	0–210	0–254	0–268	0–156	0–330
	R_L,t_	198–441	226–525	268–441	300–441	170–441	416–441
	R_L,ss_	442–525	442–525	442–525	442–525	442–525	442–525
60	R_L,i_	0–22	0–15	0–28	0–22	0–15	0–15
	R_L,t_	28–86	22–79	35–72	28–65	22–79	22–79
	R_L,ss_	100–226	86–226	79–226	72–226	86–226	86–226

**Table 2:** Normalised dissolution rate of uranium (R_L_(U)) for each regime (g m^−2^ d^−1^).

	Temperature (°C)		
	25	40	60
R_L,i_ (g m^−2^ d^−1^)			
UO_2_	(2.12 ± 0.17) × 10^−3^	(4.15 ± 0.42) × 10^−3^	(1.71 ± 0.18) × 10^−2^
147 ppm Cr	(1.80 ± 0.18) × 10^−3^	(3.60 ± 0.36) × 10^−3^	(1.83 ± 0.18) × 10^−2^
337 ppm Cr	-	(3.60 ± 0.36) × 10^−3^	(2.20 ± 0.22) × 10^−2^
554 ppm Cr	(1.44 ± 0.14) × 10^−3^	(3.13 ± 0.31) × 10^−3^	(2.46 ± 0.25) × 10^−2^
741 ppm Cr	(1.44 ± 0.14) × 10^−3^	(2.79 ± 0.28) × 10^−3^	(2.21 ± 0.20) × 10^−2^
1490 ppm Cr	(1.31 ± 0.13) × 10^−3^	(2.56 ± 0.26) × 10^−3^	(3.10 ± 0.31) × 10^−2^
R_L,t_ (g m^−2^ d^−1^)			
UO_2_	(1.10 ± 0.09) × 10^−3^	(2.45 ± 0.24) × 10^−3^	(2.99 ± 0.30) × 10^−3^
147 ppm Cr	(0.81 ± 0.08) × 10^−3^	(2.56 ± 0.23) × 10^−3^	(1.43 ± 0.14) × 10^−3^
337 ppm Cr	-	(2.04 ± 0.20) × 10^−3^	(2.50 ± 0.25) × 10^−3^
554 ppm Cr	(0.78 ± 0.08) × 10^−3^	(1.77 ± 0.18) × 10^−3^	(3.64 ± 0.36) × 10^−3^
741 ppm Cr	(0.46 ± 0.05) × 1^0−3^	(1.66 ± 0.17) × 10^−3^	(3.36 ± 0.34) × 10^−3^
1490 ppm Cr	(0.39 ± 0.04) × 10^−3^	(1.44 ± 0.14) × 10^−3^	(4.61 ± 0.46) × 10^−3^
R_L,ss_ (g m^−2^ d^−1^)			
UO_2_	-	(0.03 ± 0.01) × 10^−3^	(-1.46 ± 0.15) × 10^−3^
147 ppm Cr	(5.41 ± 0.53) × 10^−5^	(0.74 ± 0.07) × 10^−3^	(-0.64 ± 0.06) × 10^−3^
337 ppm Cr	-	(-1.57 ± 0.16) × 10^−3^	(-0.26 ± 0.03) × 10^−3^
554 ppm Cr	(5.88 ± 0.58) × 10^−5^	(-1.03 ± 0.10) × 10^−3^	(-0.68 ± 0.07) × 10^−3^
741 ppm Cr	(2.25 ± 0.23) × 10^−5^	(-2.07 ± 0.21) × 10^−3^	(-0.18 ± 0.02) × 10^−3^
1490 ppm Cr	(3.89 ± 0.40) × 10^−5^	(-1.47 ± 0.15) × 10^−3^	(-1.14 ± 0.11) × 10^−3^
R_L,gb_ (g m^-2^ d^-1^)			
UO_2_			
147 ppm Cr	(1.05 ± 0.11) × 10^−3^	-	-
337 ppm Cr	-	-	-
554 ppm Cr	(1.02 ± 0.10) × 10^−3^	-	-
741 ppm Cr	(0.95 ± 0.10) × 10^−3^	-	-
1490 ppm Cr	(0.42 ± 0.04) × 10^−3^	-	-

Please see Supplementary Material Table 1 for corresponding data in mol L^−1^ d^−1^.

R_L,i_ (initial), R_L,t_ (transitional), R_L,ss_ (steady state), and R_L,gb_ (grain boundary effects).

### 25 °C experiment

The normalised dissolution rate of U decreased with increasing Cr content in UO_2_ dissolved at 25 °C, in both the R_L,i_ and R_L,t_ regimes (Fig. 3a, b). For example, R_L,i_ = (2.12 ± 0.17) × 10^−3^ g m^−2^ d^−1^ for undoped UO_2_ and (1.31 ± 0.13) × 10^−3^ g m^−2^ d^–1^ for 1490 ppm Cr-doped UO_2_. The corresponding dissolution rates in the R_L,t_ regime were (1.10 ± 0.09) × 10^−3^ g m^−2^ d^−1^ and (0.39 ± 0.04) × 10^−3^ g m^−2^ d^−1^ for undoped and Cr-doped UO_2_, respectively (Table 2). From these results, it is apparent that the presence of Cr in UO_2_, in solid solution with the UO_2_ matrix and/or at present as precipitates at grain boundaries, influences the dissolution behaviour of U.

Since UO_2_ dissolution is dependent on the oxidation of U^4+^ to soluble U^6+^ species, especially under the oxic conditions of the present study, it is hypothesised that preferential oxidation of Cr^2+^/Cr^3+^ allows U to maintain a reduced state through the action of a galvanic coupling effect, according to the simplified form of the redox couples in Eqs. 1 and 2.

1 $$2Cr^{2 + } + U^{6 + } \to 2Cr^{3 + } + U^{4 + } = 0.75\;V$$

2 $$2Cr^{3 + } + U^{6 + } \to 2Cr^{6 + } + U^{4 + } = - 1.033\;V$$

Clearly, the Cr^3+^-induced reduction of U^6+^ to U^4+^ is thermodynamically unfavourable (Eq. 2), meaning that the presence of Cr^2+^ species in the UO_2_ matrix is responsible for the decreased U dissolution rate. Increased Cr^2+^ content as a function of Cr doping would explain why the galvanic coupling effect is most pronounced in the more highly Cr-doped UO_2_, in the R_L,i_ and R_L,t_ regimes.

During the latter stages of the dissolution experiment, in the solubility-controlled R_L,ss_ regime, a steady state was reached for U leached from Cr-doped UO_2_ at 25 °C (Fig. 2a). Importantly, in this steady state regime, there appeared to be no significant difference in the normalised dissolution rate of U as a function of Cr content (Fig. 4a). However, the absence of steady-state conditions for the undoped UO_2_ signifies an effect of Cr on the thermodynamics of U dissolution.

Since no Cr was measured in solution, it is assumed that sufficient Cr^2+^ remained within the doped UO_2_ to promote the galvanic coupling effect during the earlier regimes of dissolution; however, this process clearly no longer controlled the dissolution rate for the R_L,ss_ and R_L,gb_ regimes (Fig. 4a, b, respectively). It is possible that, at later time points, the Cr^2+^ involved in this mechanism was fully oxidised and, therefore, had no further potential for galvanic coupling. Alternatively, solution saturation of U may have hindered further dissolution. The measured molar concentration of U in the R_L,ss_ regime was ~0.6–1.8 × 10^−6^ mol L^−1^ (Supporting Information, Fig. 1), which is below, but close to, the solubility limit of schoepite, reported to be around 10^−5^ mol L^−1^ under similar conditions^33,37^. As such, the solution was close to saturation with respect to U-containing secondary phases, reducing the thermodynamic driver for further dissolution of U. It was not possible to identify any secondary phases precipitated at the surface of the 25 °C pellets, even after 761 days of dissolution; therefore, the former explanation, that Cr^2+^ was fully oxidised, is tentatively favoured.

Beyond 616 days of dissolution at 25 °C, the role of grain boundary dissolution increased the U dissolution rate from ~10^−4^ g m^−2^ d^−1^ in the R_L,ss_ regime to ~10^−3^ g m^−2^ d^−1^ in the R_L,gb_ regime (Fig. 4b and Table 2).

### 40 °C experiment

The normalised dissolution rate of U, at 40 °C, in the R_L,i_ (Fig. 3c) regime also decreased as a function of Cr content, from (4.15 ± 0.42) × 10^−3^ g m^−2^ d^−1^ to (2.56 ± 0.26) × 10^−3^ g m^−2^ d^−1^ for undoped UO_2_ and the highest Cr-doped UO_2_ material, respectively. Similar behaviour was observed in the R_L,t_ regime (Fig. 3d), with rates of (2.45 ± 0.24) × 10^−3^ g m^−2^ d^−1^ for undoped UO_2_ and (1.44 ± 0.14) × 10^−3^ g m^−2^ d^−1^ for the highest concentration of Cr-dopant. It follows that the galvanic coupling effect of Cr^2+^ in UO_2_, as described for the 25 °C experiment, also occurs at 40 °C.

**Fig. 5:**
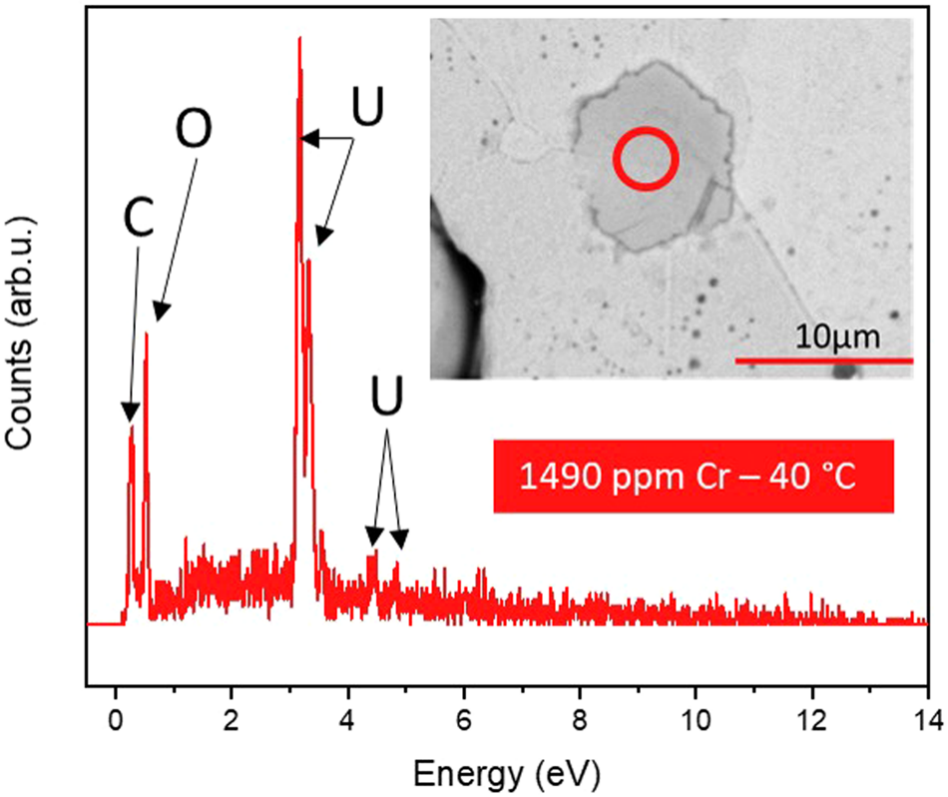
SEM-EDX analysis of surface precipitate with plate morphology. Acquired from a sample of 1490 ppm Cr-doped UO_2_ (prepared at 1700 °C) and dissolved at 40 °C in bicarbonate solution for 525 days.

A steady-state regime was reached for all experiments at 40 °C after 442 days (Table 2), where the R_L,ss_ was (0.03 ± 0.01) × 10^−3^ g m^−2^ d^−1^ for UO_2_ and (0.74 ± 0.10) × 10^−3^ g m^−2^ d^−1^ for 147 ppm Cr. The molarity values of U (3.7 × 10^−6^ mol L^−1^ and 3.4 × 10^−6^ mol L^−1^, respectively) were close to the solubility limit of U in saline, bicarbonate solution under oxic conditions, meaning that the solution was close to saturation with respect to U-containing secondary phases. The absolute normalised dissolution rate of U for UO_2_ doped with higher concentrations of Cr were negative, concurrent with the precipitation of U-bearing secondary phases, identified via SEM imaging of the material surfaces post-dissolution (Fig. 5).

### 60 °C experiment

At 60 °C, the undoped UO_2_ exhibited a lower N_L_(U) than the Cr-doped UO_2_ (Fig. 2c) and the reverse trend in normalised dissolution rate of U was found for R_L,i_ (Fig. 3e) and R_L,t_ (Fig. 3f) when compared with 25 and 40 °C, i.e. the dissolution of U increased with increasing Cr content. It has previously been shown that bicarbonate-promoted dissolution of UO_2_, where bicarbonate ligands bind to the initially oxidised UO_2_ surface, is strongly dependent on temperature, with dissolution rates increasing in the range of 10 to 60 °C^38^. This is because the activation energy of UO_2_ surface oxidation is greater than that of the surface attachment of the bicarbonate ligand^38^. Therefore, given the higher temperature of this particular experiment, we postulate that the role of bicarbonate in the dissolution process is more significant than the galvanic coupling effect of Cr^2+^.

**Fig. 6:**
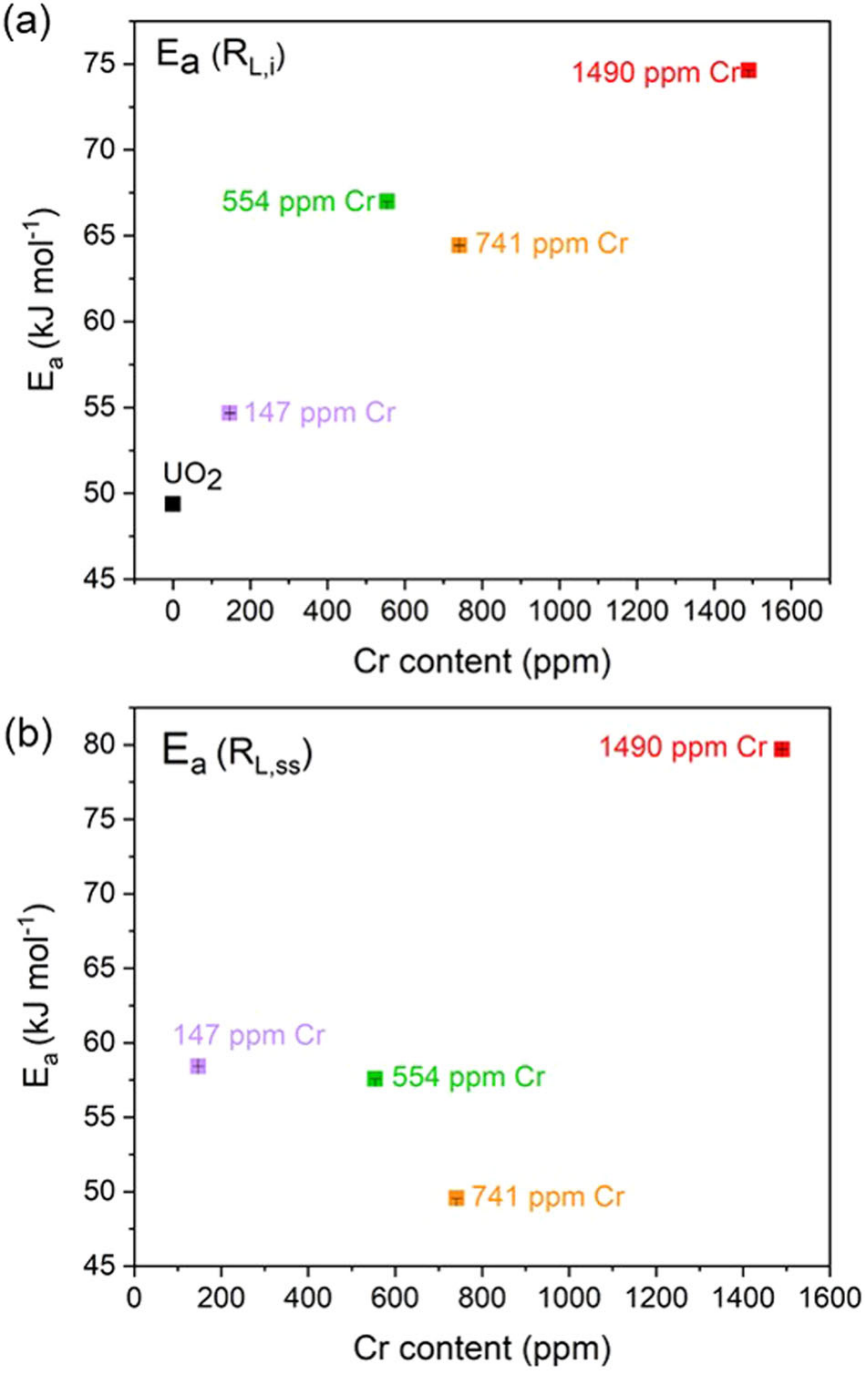
Apparent activation energy of undoped and Cr-doped UO_2_ determined at 25, 40 and 60 °C. **a** Showing activation energies derived from rates in the initial dissolution rate regime (R_L,i_) and **b** During the steady-state dissolution regime (R_L,ss_). Error bars (smaller than data points but shown within points) represent one standard deviation of triplicate measurements.

Calculations of the apparent activation energy (E_a_) (see method section for details), determined for undoped and Cr-doped UO_2_ at 25, 40 and 60 °C using the R_L,i_ dissolution rates are shown in Fig. 6a. In agreement with published data for UO_2_ dissolution in bicarbonate solution, the undoped UO_2_ gave an E_a_ value of 49.40 ± 0.04 kJ mol^−1^, consistent with a dissolution mechanism that proceeds via surface-controlled reactions^38^. As the Cr content is increased, the activation energy significantly increased, reaching (78.90 ± 0.01) kJ mol^−1^ for the highest concentration of Cr-dopant. This suggests that, in the initial stage of dissolution (i.e. without the effects of solution saturation), Cr influences surface-controlled dissolution reactions, consistent with the hypothesised galvanic coupling effect, even in bicarbonate solutions.

**Fig. 7:**
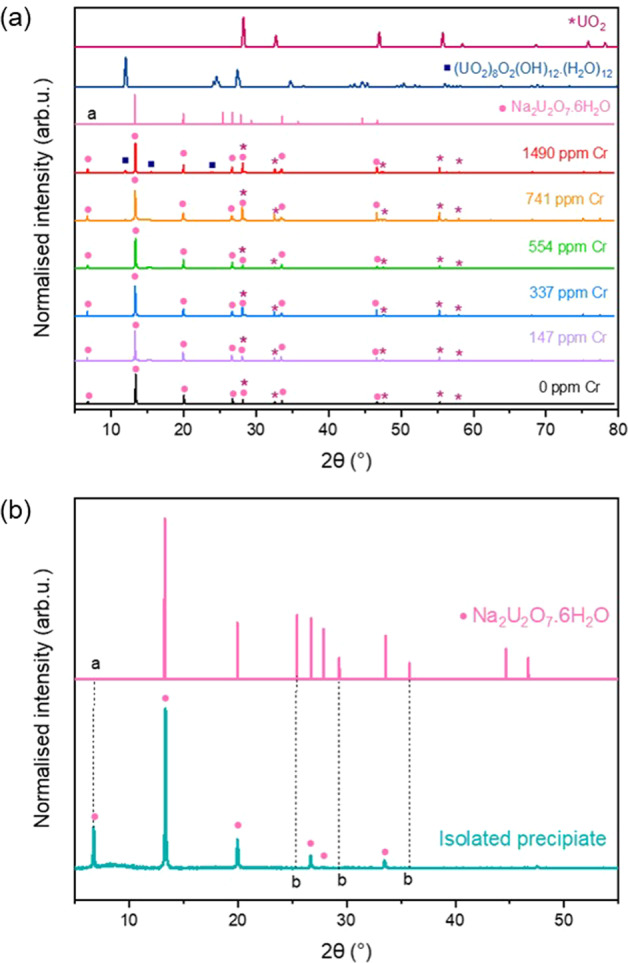
XRD patterns of precipitated phases. **a** XRD pattern of Cr-doped UO_2_ surfaces dissolved at 60 °C in bicarbonate solution after 226 days of dissolution in comparison to diffraction patterns of UO_2_, Na_2_U_2_O_7_.6H_2_O, and ((UO_2_)_8_O_2_(OH)_12_(H_2_O)_12_) and **b** XRD pattern of isolated precipitate phase removed from the dissolved surface. Point (**a**) refers to the unpublished peak at low angles of 2θ, and points (**b**) represent absent reflections due to the preferred orientation of platy phases at the Cr-doped UO_2_ surface.

The steady-state regime was reached after 100 days of dissolution at 60°C for undoped UO_2_ and after 79–86 days for Cr-doped UO_2_ (Table 1). The absolute normalised dissolution rates of U were negative (Table 2), indicating the formation of U-bearing secondary phases, which were more prominent at 60 than 40 °C and, as such, identifiable by XRD (Fig. 7). XRD diffraction patterns were consistent between duplicate pellets; therefore, only one representative pattern of each is shown.

**Fig. 8:**
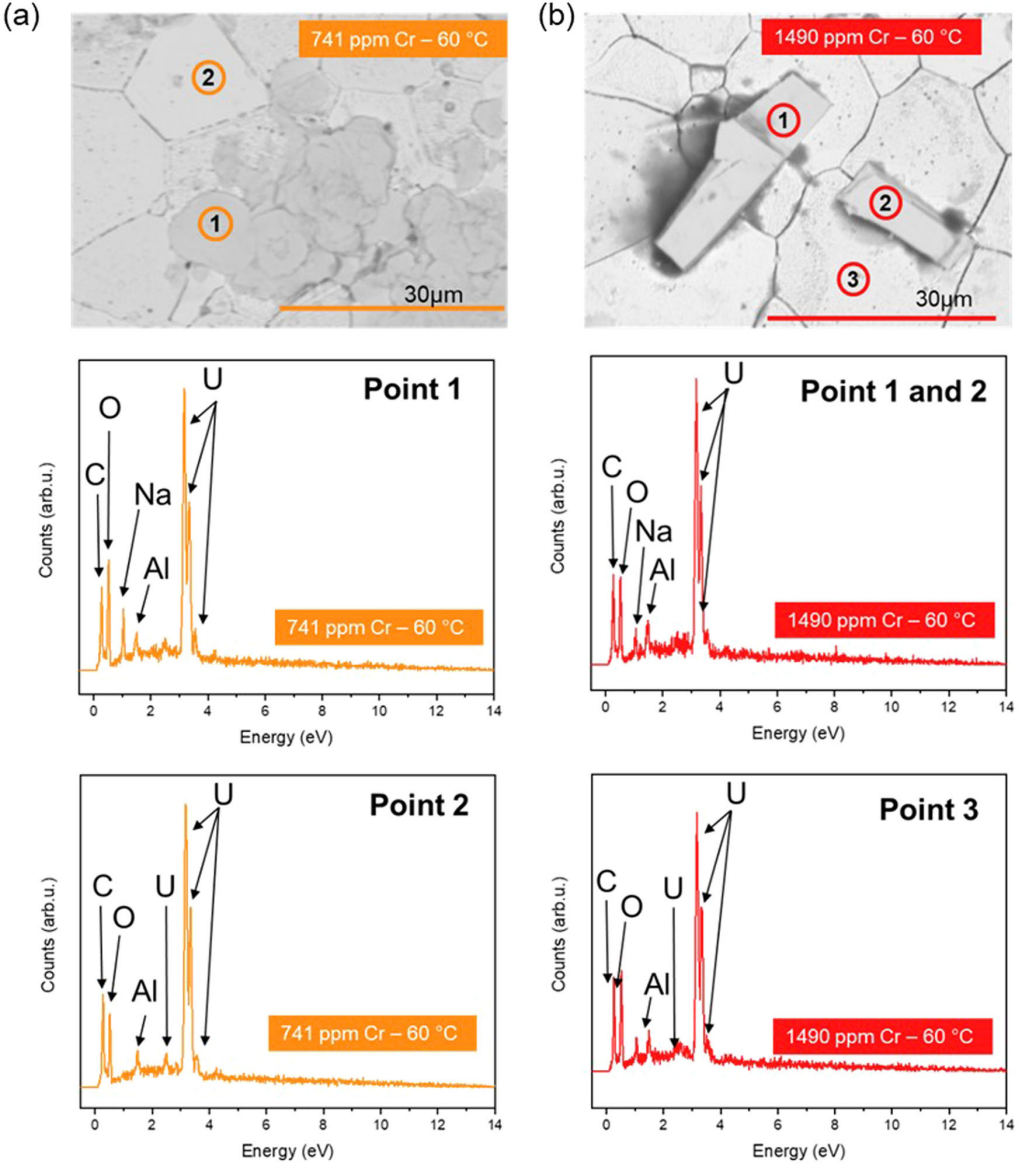
SEM images and EDX point spectra of precipitate phases observed in Cr-doped UO_2_ dissolved at 60 °C in bicarbonate solution for 226 days. **a** 741 ppm Cr-doped UO_2_ plate-like precipitate, identified as Na_2_U_2_O_7_·6H_2_O and **b** 1490 ppm Cr-doped UO_2_ chip-like precipitate, identified as schoepite ((UO_2_)_8_O_2_(OH)_12_·(H_2_O)_12_).

**Fig. 9:**
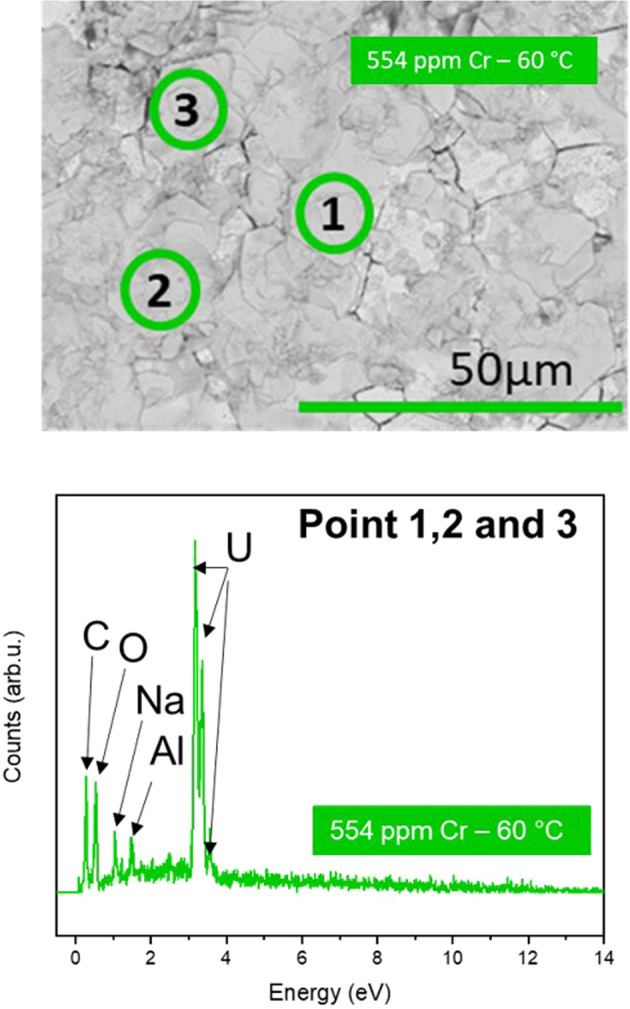
Surface characterisation of 554 ppm Cr-doped UO_2_ surface dissolved at 60 °C in bicarbonate solution for 226 days. SEM image of plate-like precipitate and corresponding EDX point spectrum representative of Points 1, 2 and 3.

Two distinct precipitate morphologies were identified: a plate-like phase (Figs. 8a, 9), present on all dissolved materials (doped and undoped UO_2_); and an angular chip-shaped phase (Fig. 8b), visible only at the surface of the highest concentrations of Cr dopant. EDX point analysis of each precipitate confirmed that both phases comprised U, O and Na (Figs. 8a, 9). A darker phase was also identified in some areas, containing Na and Cl (Supporting Data, Fig. 2a; Point 1). This phase appeared not to contribute to the plate-like or chip-like morphologies in EDX elemental mapping (Supporting Information, Fig. 2b) and was likely remnant NaCl from the solution.

XRD data were compared with UO_2_^39^, and analysis of the plate-like precipitate matched with most of the reflections of hydrated Na-diuranate^40^, Na_2_U_2_O_7·_6H_2_O (Fig. 7b). This identification is tentatively corroborated by the EDX measured stoichiometry U:Na ratio of 1:1 (Table 3, taken from the precipitate layer with greatest apparent thickness). An additional reflection at ~6.7° 2θ (Fig. 7a, b; Point a), not previously published in the literature due to the restricted angular range used, corresponds to a d spacing of ~13.1 Å, which is in agreement with the postulated structure of Na_2_U_2_O_7·_6H_2_O^40^. Absent peaks at higher angles of 2θ (Fig. 7b; Points labelled b) can be attributed to preferred orientation arising from the flat, plate-like morphology. Reflections consistent with schoepite ((UO_2_)_8_O_2_(OH)_12_·(H_2_O)_12_) were also present in UO_2_ doped with the highest concentration of Cr (1490 ppm Cr, Fig. 7a)^41^.

**Table 3:** Qualitative EDX point spectrum analysis of U precipitate phases.

SEM image reference	Sample	Morphology	U (at.% ± 0.1)	O (at.% ± 0.1)	Na (at.% ± 0.1)
Fig. 8a Point 1	554 ppm Cr	Plate	20.7	64.3	15.0
Fig. 8a Point 2	554 ppm Cr	Plate	18.8	63.7	17.5
Fig. 8a Point 3	554 ppm Cr	Plate	17.3	67.4	15.3
Fig. 8a Point 4*	554 ppm Cr	Plate	18.3	62.5	19.2
Fig. 8a Point 5*	554 ppm Cr	Plate	18.4	65.6	18.1
Fig. 8a Point 6*	554 ppm Cr	Plate	18.0	65.7	16.3
Fig. 8a Point 7*	554 ppm Cr	Plate	17.3	67.4	15.3
Fig. 8a Point 8*	554 ppm Cr	Plate	17.4	65.6	17.0
Fig. 8a Point 9*	554 ppm Cr	Plate	15.2	67.0	17.8
Fig. 8a Point 10*	554 ppm Cr	Plate	16.7	68.0	15.3
Average (at% ± 0.1)			17.8	65.7	16.7
Ratio U/Na			1.07		

Due to EDX probe depth, measurements were taken only on precipitates deemed to be thicker than 1 μm.

*not labelled in Fig. 8a.

The apparent activation energy in the R_L,ss_ regime (Fig. 6b), where solution saturation influences the dissolution behaviour, was shown to remain within the same range as the E_a_ for R_L,i_ regime (49.60 ± 0.01 to 79.70 ± 0.01 kJ mol^−1^); however, there was no clear trend between E_a_ and Cr content. This supports the hypothesis that at later stages of dissolution, when the solubility limit of U in bicarbonate solution has been reached, the effect of Cr is negligible. Interestingly, the E_a_ remains unchanged for UO_2_ doped with the highest concentration of Cr, which gave an E_a_ of R_L,i_ = 74.63 ± 0.01 kJ mol^−1^ and R_L,ss_ = 79.68 ± 0.02 kJ mol^−1^. To fully understand the mechanistic behaviour, a wider range of Cr-doped UO_2_ concentrations should be investigated in future.

### Effect of grain size on the dissolution of undoped and Cr-doped UO_2_

To investigate the role of grain size on dissolution, undoped and Cr-doped UO_2_, prepared in a reducing atmosphere, were subject to heat treatment at different sintering temperatures of 1400, 1500, 1600, and 1700 °C. A clear increase in grain size with increasing sintering temperature was observed, from ~2–14 μm (Fig. 1a).

Complete digestion of these sintered materials showed that the Cr content was below the expected, nominal, Cr concentration (Fig. 1b) due to volatilisation^31,32^. The extent of volatilisation was much greater for materials sintered at temperatures of ≤1600 °C when compared with those at 1700 °C. It is understood that the solubility of Cr increases with increasing temperature^26^; therefore, at lower sintering temperatures, where less Cr is incorporated in the UO_2_ lattice, a greater loss due to volatilisation was observed. The greater extent of volatilisation of Cr at the lower sintering temperatures is corroborated by the measured densification rate, determined via Archimedes density measurements (Fig. 1c). The densification rate was highest for materials sintered at 1700 °C (~98%), as expected, but only reached ~91–96% in materials sintered ≤1600 °C. Since Cr incorporation into the lattice is attributed to increased densification of Cr-doped UO_2_^32^, it follows that when there is less Cr in the lattice, at lower sintering temperatures, a reduced densification rate would be observed. As such, neither the Cr content (Fig. 1b), nor the density (Fig. 1c), were consistent between pellets, i.e. grain size was not the only variable during the study.

**Fig. 10:**
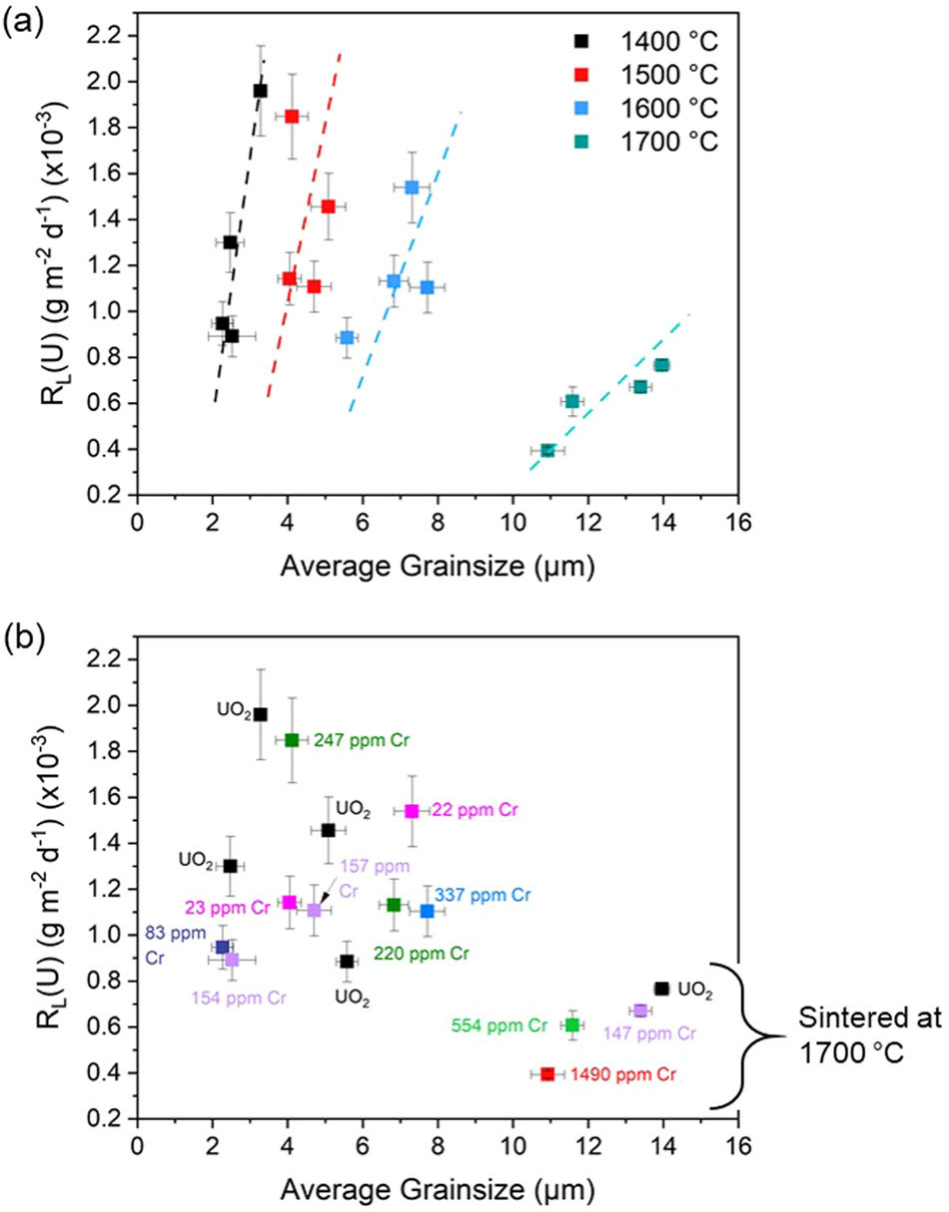
The normalised dissolution rate (R_L_(U)) of undoped and Cr-doped UO_2_ as a function of grain size. Data were derived from experiments performed at 25 °C in a bicarbonate solution for 100 days. **a** Showing the relationship between sintering temperature, average grain size and R_L_(U) and **b** The relationship between Cr content, average grain size and R_L_(U). Error bars represent one standard deviation of triplicate measurements.

These materials were subject to dissolution in simulant groundwater solution (1 mM NaHCO_3_ + 19 mM NaCl) at 25 °C for 100 days (assumed to be the R_L,i_ regime, based on data in Table 2). For materials sintered at 1700 °C, which display a high rate of densification (~97%, Fig. 1c), there is an obvious correlation between average grain size and the normalised dissolution rate of U (Fig. 10a, b). This correlation is also evident for materials sintered at lower temperatures (Fig. 10a). This result agrees with the hypothesis that a reduction in grain size, and therefore the quantity of grain boundaries, results in fewer energetically reactive surface sites for initiation of dissolution^17,18^. By observation of the gradient of R_L_(U) within each set of materials sintered at the different temperatures, it is clear as the sintering temperature is increased, the change in grain size has less of an effect on the normalised dissolution rate, *i.e*., the gradient becomes more shallow with increasing temperatures, most probably due to the level of densification attained.

When the measured Cr content of each material is considered (Fig. 10b) at sintering temperatures of ≤1600 °C, there is no relationship between the dissolution rate and the Cr content. We postulate that variability in density between pellets may give rise to this behaviour. Further study, using Cr-doped UO_2_ materials prepared under conditions designed to stimulate enhanced grain growth while ensuring constant density and Cr content, is required to fully assess the effects of each variable in complete isolation.

## Methods

### Cr-doped and undoped UO_2_ preparation

A wet chemical, nitrate precipitation method was used to synthesise both Cr-doped and undoped UO_2_ materials at the HADES National Nuclear User Facility^42^. Nominal concentrations of Cr dopant were chosen, above and below the proposed solubility limit of Cr in UO_2_ (700–1200 ppm)^26–29^. Uranium (VI) nitrate hexahydrate in solution (UO_2_(NO_3_)_2_·6H_2_O, The British Drug House (BDH). B.D.H Laboratory Chemicals Division, >98%, 0.3 mol L^−1^) was mixed with various amounts of chromium (III) nitrate nonahydrate in solution (Cr(NO_3_)_3_·9H_2_O, Sigma Aldrich, 99.99%, 1.6 mol L^−1^) and precipitated at room temperature using concentrated ammonium hydroxide solution (NH_4_OH, Sigma Aldrich, 28–30% NH_3_ in H_2_O 5 mol L^−1^). The pH was monitored to reach between pH 8–10 for the successful co-precipitation of U and Cr, confirmed by Inductively Coupled Plasma Optical Emission Spectroscopy (ICP-OES, Thermo Fisher iCAP Duo6300) of the supernatants, where 99.9% precipitation for each element was achieved. The yellow precipitate was washed in deionised water, vacuum filtered and dried overnight at 90 °C to eliminate any remaining hydroxide. The resultant precursor powder was converted to oxide *via* thermal treatment at 750 ˚C for 4 h under a reducing (95% N_2(g)_–5% H_2(g)_) atmosphere, followed by dry milling at 35 Hz for 15 min to increase homogeneity and powder reactivity. Pellets of 6 mm were uniaxially pressed at 2.5 tonnes using a stainless steel die, and sintered for 8 h in a reducing (95% N_2(g)_–5% H_2(g)_) atmosphere. A range of sintering temperatures, of 1400, 1500, 1600 and 1700 °C, were used to control the microstructure (grain size). All surfaces were ground using SiC paper, polished to 1 μm using diamond suspension and thermally etched at ~85–90% sintering temperature to ensure an equal surface finish for all materials and to reveal the grain structure for characterisation.

### Cr-doped and undoped UO_2_ characterisation

Confirmation of the UO_2_ single phase was carried out via X-ray diffraction (XRD) using a Bruker D2 Phaser diffractometer utilising a Cu Kα source from 10° to 100° 2θ with a step size of 0.02° and a step time of 2 s. Geometric densities of sintered pellets were calculated using the average geometry, measured using calibrated digital callipers, and average mass, measured using a calibrated five-point balance, to determine the geometric specific surface area of each pellet. Archimedes density was also determined for each individual pellet prior to dissolution, presented as an average of 10 measurements. The microstructures of the Cr-doped and undoped UO_2_ were characterised by scanning electron microscopy (SEM) using a Hitachi TM3030 SEM operating with an accelerating voltage of 15 kV. Images were taken at 500x magnification across five points of each pellet and the average grain area of ~500 grains was measured using ImageJ.

The total Cr content within the sintered materials was assessed by a complete digest in concentrated nitric acid (2 M HNO_3_). Pellets were crushed using a pestle and mortar and ~20 mg of powder was completely dissolved, under temperature and stirring, in 5 mL HNO_3_. The solutions were then measured for Cr concentration by Inductively Coupled Plasma Mass Spectroscopy (ICP-MS, Thermo Fisher single quadrupole iCAP RQ). Grain size and density were quantified as a function of both Cr content and sintering temperature.

### Oxic dissolution experiments

Long-term durability experiments were conducted in the PLEIADES National Nuclear User Facility, using thermally etched, sintered pellets in duplicate, which were submerged in 50 mL of simulant groundwater solution (19 mM NaCl + 1 mM NaHCO_3_ bicarbonate solution) in a PTFE container. No atmospheric control was applied throughout the experiment, which is important to note since the dissolution medium was in equilibrium with CO_2_ in the air. The pH was measured to be in the range of 7.8 to 8.2 (±0.2 pH units) for all experiments at all times.

To understand the role of Cr content on dissolution behaviour, experiments were performed in ovens at 25, 40 and 60 °C (±2 °C) on UO_2_ with increasing Cr content, sintered at 1700 °C. To establish the role of grain size on Cr-doped UO_2_ dissolution, further experiments were performed at 25 °C on a selection of Cr-concentrations sintered at 1400, 1500, 1600 and 1700 °C. Both experiments are discussed and compared to assess the influence of Cr content, temperature and microstructure on dissolution behaviour.

At specific time points, an aliquot of 2 mL of the dissolution medium was removed, filtered (0.22 µm) and diluted by a factor of 10 in 1% ultra-pure conc. HNO_3_ for analysis by ICP-MS. A fresh solution of the same volume was replaced to maintain the surface area to volume (SA/V) ratio of approximately 1.3 m^−1^. Aliquots of solution were taken on days 1, 3, 7, 14, 21, then weekly up to day 98 and then bi-weekly for the duration of each experiment, which varied depending on dissolution temperature. It should be noted that Covid-19-induced laboratory closures severely restricted the sampling points for several experiments, over a period of ~6 months. Each long-term experiment was terminated after a constant concentration of U was measured, within error, for four consecutive time points.

The concentration of U (C(U), ng L^−1^) was converted to the mass of the element in solution for each time point (m(U)(t), mg) using the volume of each aliquot (V, 2 mL) via Eq. 3. The concentration of Cr was below limits of detection (2.5 ng L^−1^ for KED and 28 ng L^−1^ for STD analysis modes) for the duration of all experiments and, therefore, could not be measured.

3 $$m\left( U \right)(t) = C\left( U \right) \times V$$

4 $$m_{{{remaining}}}\left( U \right)\left( t \right) = m_{{{initial}}} - \left[ {\frac{{m_{{\mathrm{loss}}}\left( U \right)(t)}}{{f(U)}}} \right]$$

5 $$N_L(U) = \frac{{m_{{\mathrm{loss}}}\;(U)(t)}}{{f\left( U \right) \times S_{{\mathrm{SA}}} \times m(U)(t - 1)}}$$

The cumulative mass loss over time (m_loss_(U)(t), mg) of each pellet, and the initial pellet mass (m_initial_, mg) was used to determine the percentage of mass remaining for each pellet via Eq. 4, where f(U) is the mass fraction of U in each pellet. The normalised mass loss N_L_(U) (g m^−2^) was calculated from Eq. 5 using the specific geometric surface area (S_SA,_ m^2^ g^−1^) of each pellet, while the normalised rate of U dissolution (R_L_(U), g m^−2^ d^−1^) was determined *via* the gradient of N_L_(U) as a function of time.

The dependence of the dissolution rate on temperature was assessed by determination of the activation energy (E_a_) according to the Arrhenius law, Eq. 6, where R_L_(U) was taken in the initial regime of dissolution (R_Li_, Table 1) as well as when the effects of solution saturation were observed in the steady state of U dissolution (R_Lss_, Table 1). The gradient of ln(R_L_(U)), for the intended regime, over the reciprocal absolute temperature was taken to determine the E_a_ (kJ mol^−1^) for undoped and Cr-doped UO_2_, as a function of Cr content.

6 $$R_L\left( U \right) = e^{ - E_a/{\mathrm{RT}}}$$

### Secondary phase characterisation

The presence of secondary phases, formed during dissolution, was determined via SEM/EDX using a Hitachi TM3030 SEM coupled with Bruker Quantax EDX system. XRD of the pellet surface, post-dissolution, was performed as described above, between 5° and 100° 2θ with a step size of 0.02° and a step time of 2 s. To improve diffraction pattern analysis, the precipitates were gently removed from the surfaces of pellets and analysed. XRD patterns were indexed using PDF SIEV + software. Geochemical modelling using PHREEQC-3 and the LLNL database was used to identify potential species that reached saturation limits in the dissolution.

## Supplementary information

Supplemental material

## Data Availability

The data that support the findings of this study are available from the corresponding author upon reasonable request.

## References

[1] Badley, M. & Shoesmith, D. W. *The Corrosion/Dissolution of Used Nuclear Fuel in a Deep Geologic Repository*. Report No. NWMO-TR-2022-09 (Nuclear Waste Management Organisation (NWMO), Toronto, Canada, 2022).

[2] Oversby, V. M. *Uranium Dioxide, SIMFUEL and Spent Fuel Dissolution Rates – A Review of Published Data.* Report No. TR-99-22 (Svensk Karnbranslehantering AB (SKB), Stockholm, Sweden, 1999).

[3] Shoesmith DW (2000). Fuel corrosion processes under waste disposal conditions. J. Nucl. Mater..

[4] Bruno J, Ewing RC (2006). Spent nuclear fuel. Elements.

[5] Guillet, J. L. & Guerin, Y. *Nuclear Fuels*. Report No. ISBN 978-2-281-11345-7 (Commissariat à l’énergie atomique (CEA), Gif-sur-Yvette Cedex, France, 2009).

[6] Massih, A. R. *An Evaluation of High-Temperature Creep of Zirconium Alloys: Data versus Models. Swedish Radiation Safety Authority Report*. Report No. 2014:20 ISSN 2000-0456 (Strålsäkerhetsmyndigheten (SSM), Stockholm (Sweden), 2014).

[7] Assmann H, Dörr W, Peehs M (1986). Control of UO_2_ microstructure by oxidative sintering,. J. Nucl. Mater..

[8] He H, Qin Z, Shoesmith DW (2010). Characterising the relationship between hyperstoichiometry, defect structure and local corrosion kinetics of uranium dioxide. Electrochim. Acta.

[9] Smith H (2022). Cr^2+^ solid solution in UO_2_ evidenced by advanced spectroscopy. Comm. Chem..

[10] Cachoir C, Mennecart T, Lemmens K (2021). Evolution of the uranium concentration in dissolution experiments with Cr(Pu)-doped UO_2_ in reducing conditions at SCK CEN. MRS Adv..

[11] Nilsson K, Roth O, Jonsson M (2017). Oxidative dissolution of ADOPT compared to standard UO_2_ fuel. J. Nucl. Mater..

[12] Casella A, Hanson B, Miller W (2016). The effect of fuel chemistry on UO_2_ dissolution. J. Nucl. Mater..

[13] Kim J-G (2001). Effect of a trivalent dopant, Gd^3+^, on the oxidation of uranium dioxide. J. Nucl. Mater..

[14] Liu N (2017). Influence of Gd doping on the structure and electrochemical behaviour of UO_2_. Electrochim. Acta.

[15] Razdan M, Shoesmith DW (2014). Influence of trivalent-dopants on the structural and electrochemical properties of uranium dioxide (UO_2_). J. Electrochem. Soc..

[16] Liu N, He H, Noël JJ, Shoesmith DW (2017). The electrochemical study of Dy_2_O_3_ doped UO_2_ in slightly alkaline sodium carbonate/bicarbonate and phosphate solutions. Electrochim. Acta.

[17] Corkhill CL (2014). Contribution of energetically reactive surface features to the dissolution of CeO_2_ and ThO_2_ analogues for spent nuclear fuel microstructures. Appl. Mater. Interfaces.

[18] Corkhill CL (2016). Role of microstructure and surface defects on the dissolution kinetics of CeO_2_, a UO_2_ fuel analogue. Appl. Mater. Interfaces.

[19] Claparede L (2011). Influence of crystallisation state and microstructure on the chemical durability of cerium-neodymium mixed oxides. Inorg. Chem..

[20] Horlait D (2014). Environmental SEM monitoring of Ce_1−x_Ln_x_O_2−x/2_ mixed-oxide microstructural evolution during dissolution. J. Mater. Chem. A.

[21] Szenknect S (2012). Kinetics of structural and microstructural changes at the solid/solution interface during dissolution of cerium(IV)-neodymium(III) oxides. J. Phys. Chem. C..

[22] Myllykylä E (2015). Solution composition and particle size effects on the dissolution and solubility of a ThO_2_ microstructural analogue for UO_2_ matrix of nuclear fuel. Radiochim. Acta.

[23] Cordara T (2019). Microstructural evolution of UO_2_ pellets containing metallic particles of Ru, Rh and Pd during dissolution in nitric acid solution: 3D-ESEM monitoring. Hydrometallurgy.

[24] Claparede L (2015). Dissolution of Th_1‑x_U_x_O_2_: effects of chemical composition and microstructure. J. Nucl. Mater..

[25] Kashibe S, Une K (1998). Effect of additives (Cr_2_O_3_, Al_2_O_3_, SiO_2_, MgO) on diffusional release of ^133^Xe from UO_2_ fuels. J. Nucl. Mater..

[26] Bourgeois L, Dehaudt P, Lemaignan C, Hammou A (2001). Factors governing microstructure development of Cr_2_O_3_-doped UO_2_ during sintering. J. Nucl. Mater..

[27] Milena-Pérez A (2021). Raman spectroscopy coupled to principle component analysis for studying UO_2_ nuclear fuels with different grain sizes due to the chromia addition. J. Nucl. Mater..

[28] Cardinaels T (2012). Chromia doped UO_2_ fuel: investigation of the lattice parameter. J. Nucl. Mater..

[29] Kuri G (2014). Local atomic structure of chromium bearing precipitates in chromia doped uranium dioxide investigate by combined micro-beam X-ray diffraction and absorption spectroscopy. J. Nucl. Mater..

[30] Riglet-Martial C (2014). Thermodynamics of chromium in UO_2_ fuel: a solubility model. J. Nucl. Mater..

[31] Finkeldei, S. C. et al. *Synthesis and Characterization of UO*_*2*_*Feedstocks Containing Controlled Dopants.* Report No. M3FT-19OR02021075 (Oak Ridge National Laboratory, Oak Ridge Tennessee (USA), 2019).

[32] Peres V (2012). High temperature chromium volatilisation from Cr_2_O_3_ powder and Cr_2_O_3_-doped pellets in reducing atmospheres. J. Nucl. Mater..

[33] Ollila, K. & Ahonen, L. *Solubilities of Uranium for TILA-99* Report No. 98-13 Svensk Kärnbränslehantering AB, Stockhom (Sweden), (1998).

[34] Cordara T (2017). Kinetics of dissolution of UO_2_ in nitric acid solutions: a multi-parametric study of the non-catalysed reaction. J. Nucl. Mater..

[35] Bertolotto S (2020). Effect of surface orientation on dissolution rate and surface dynamics of UO2 single crystals in nitric acid. Corros. Sci..

[36] Podor RL (2019). 3D-SEM height map series to monitor materials corrosion and dissolution. Mater. Charact..

[37] Ollila, K. *Dissolution of Unirradiated UO*_*2*_*Fuel in Synthetic Groundwater – Progress Report ’97*. Report No. 98-06 (Svensk Kärnbränslehantering AB, Stockholm (Sweden), 1998).

[38] De Pablo J (1999). The oxidative dissolution mechanism of uranium dioxide. I. The effect of temperature in hydrogen carbonate medium. Geochim. Cosmochim. Acta.

[39] Desgranges G (2009). Neutron diffraction study of the in situ oxidation of UO_2_. Inorg. Chem..

[40] Chernorukov NG, Nipruk OV, Kostrova EL (2016). Synthesis and study of sodium uranate Na_2_U_2_O_7_·6H_2_O and of products of its dehydration and thermal decomposition. Radiochemistry.

[41] Finch RJ, Cooper MA, Hawthorne FC, Ewing RC (1996). Distinguishing among schoepite, [(UO_2_)_8_O_2_(OH)_12_](H_2_O)_12_, and related minerals by X-ray powder diffraction. Can. Mineral.,.

[42] Hyatt NC (2020). The HADES facility for high activity decommissioning engineering and science: part of the UK National Nuclear user facility. IOP Conf. Ser. Mat. Sci..

