# Supplementary material for: Oxidative dissolution of Cr-doped UO2 nuclear fuel
Source: Npj Mater Degrad. 2023 Apr 7;7(1):25. doi: 10.1038/s41529-023-00347-4 (PMC10079487; doi:10.1038/s41529-023-00347-4)
Supplement: Supplementary file 1 — Supplemental material [file 41529_2023_347_MOESM1_ESM.pdf]

Oxidative dissolution of Cr-doped UO_2 fuel: Supporting Information

Hannah Smith, Théo Cordara, Clemence Gausse, Sarah E. Pepper, Claire L. Corkhill*

NucleUS Immobilisation Science Laboratory, Department of Materials Science and Engineering, The University of Sheffield, UK

*c.corkhill@sheffield.ac.uk

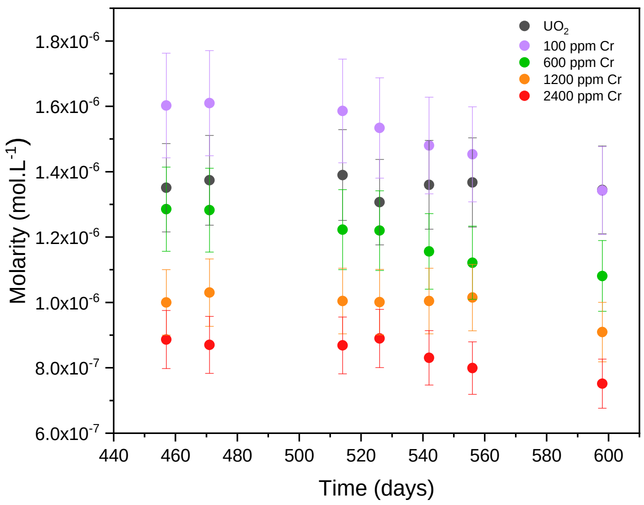

Supplementary Figure 1. The concentration of U (mol L^{-1}), for Cr-doped UO_2 in the $R_{L,SS}$ regime of dissolution, at 25 °C in bicarbonate solution. Error bars represent one standard deviation of triplicate measurements.

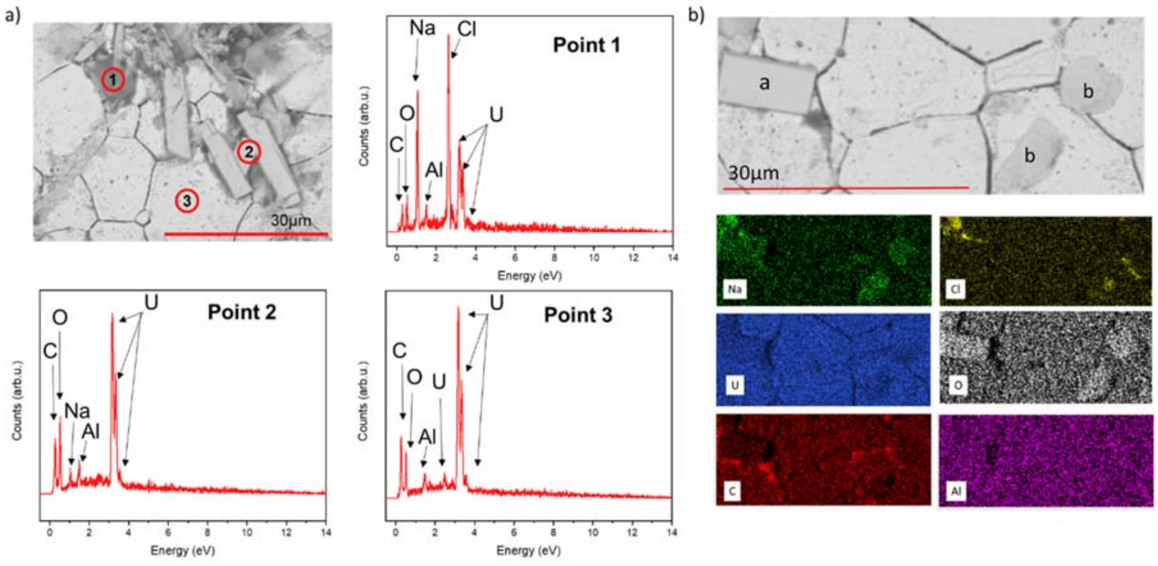

Supplementary Figure 2. EDX analysis of 1490 ppm Cr-doped sampled surface dissolved at 60 °C in bicarbonate solution after 226 days of dissolution. **a** EDX point spectra; and **b** elemental mapping showing the chip-like phase identified as schoepite ($(UO_2)_8O_2(OH)_{12}\cdot(H_2O)_{12}$) (Point a) and the plate-like phase identified as $Na_2U_2O_7\cdot 6H_2O$ (Point b)

Supplementary Table 1. Normalised dissolution rate of uranium ($R_L(U)$) for each regime, in mol L^{-1} d^{-1}. See Table 2 in the main text for corresponding data in g m^{-2} d^{-1}. Errors represent one standard deviation of triplicate measurements.

	Temperature (°C)		
	25	40	60
$R_{L,i}$ (mol L^{-1} d^{-1})			
UO_2	(4.48 ± 0.50) x 10^{-9}	(1.38 ± 0.14) x 10^{-8}	(6.18 ± 0.60) x 10^{-8}
147 ppm Cr	(5.10 ± 0.51) x 10^{-9}	(1.03 ± 0.10) x 10^{-8}	(11.6 ± 1.16) x 10^{-8}
337 ppm Cr	-	(1.02 ± 0.10) x 10^{-8}	(6.94 ± 0.70) x 10^{-8}
554 ppm Cr	(4.35 ± 0.44) x 10^{-9}	(0.88 ± 0.10) x 10^{-8}	(8.58 ± 0.86) x 10^{-8}
741 ppm Cr	(3.76 ± 0.38) x 10^{-9}	(0.88 ± 0.10 x 10^{-8}	(6.68 ± 0.70) x 10^{-8}
1490 ppm Cr	(3.77 ± 0.38) x 10^{-9}	(1.00 ± 0.10 x 10^{-8}	(12.0 ± 1.20) x 10^{-8}
$R_{L,t}$ (mol L^{-1} d^{-1})			
UO_2	(1.65 ± 0.17) x 10^{-9}	(0.51 ± 0.05) x 10^{-8}	(0.34 ± 0.03) x 10^{-8}
147 ppm Cr	(2.55 ± 0.26) x 10^{-9}	(0.39 ± 0.04) x 10^{-8}	(0.14 ± 0.01) x 10^{-8}
337 ppm Cr	-	(0.48 ± 0.05) x 10^{-8}	(0.12 ± 0.01) x 10^{-8}
554 ppm Cr	(2.42 ± 0.24) x 10^{-9}	(0.41 ± 0.04) x 10^{-8}	(0.33 ± 0.03) x 10^{-8}
741 ppm Cr	(1.14 ± 0.10) x 10^{-9}	(0.43 ± 0.04) x 10^{-8}	(0.36 ± 0.04) x 10^{-8}
1490 ppm Cr	(0.94 ± 0.10) x 10^{-9}	(0.36 ± 0.04) x 10^{-8}	(0.74 ± 0.07) x 10^{-8}
$R_{L,ss}$ (mol L^{-1} d^{-1})			
UO_2	-	(-0.21 ± 0.02) x 10^{-8}	(-0.63 ± 0.06) x 10^{-8}
147 ppm Cr	(1.01 ± 0.10) x 10^{-9}	(0.04 ± 0.01) x 10^{-8}	(-0.36 ± 0.04) x 10^{-8}
337 ppm Cr	-	(-0.48 ± 0.05) x 10^{-8}	(-0.25 ± 0.03) x 10^{-8}
554 ppm Cr	(0.62 ± 0.10) x 10^{-9}	(-1.11 ± 0.11) x 10^{-8}	(-0.45 ± 0.05) x 10^{-8}
741 ppm Cr	(0.35 ± 0.04) x 10^{-9}	(-1.85 ± 0.20) x 10^{-8}	(-0.37 ± 0.04) x 10^{-8}
1490 ppm Cr	(0.16 ± 0.02) x 10^{-9}	(-3.01 ± 0.30) x 10^{-8}	(-0.58 ± 0.06) x 10^{-8}
$R_{L,gb}$ (mol L^{-1} d^{-1})			
UO_2	-		
147 ppm Cr	(2.88 ± 0.30) x 10^{-9}	-	-
337 ppm Cr	-	-	-
554 ppm Cr	(2.95 ± 0.30) x 10^{-9}	-	-
741 ppm Cr	(2.71 ± 0.30) x 10^{-9}	-	-
1490 ppm Cr	(0.94 ± 0.10) x 10^{-9}	-	-

$R_{L,i}$ (initial); $R_{L,t}$ (transitional) $R_{L,ss}$ (steady state); and $R_{L,gb}$ (grain boundary effects)
